# Measurement of MMP-9 and -12 degraded elastin (ELM) provides unique information on lung tissue degradation

**DOI:** 10.1186/1471-2466-12-34

**Published:** 2012-07-20

**Authors:** Helene Skjøt-Arkil, Rikke E Clausen, Quoc Hai Trieu Nguyen, Yaguo Wang, Qinlong Zheng, Fernando J Martinez, Cory M Hogaboam, Meilan Han, Lloyd B Klickstein, Martin R Larsen, Arkadiusz Nawrocki, Diana J Leeming, Morten A Karsdal

**Affiliations:** 1 Nordic Bioscience A/S, Herlev Hovedgade 207, DK-2730, Herlev, Denmark; 2 Nordic Bioscience Beijing, Beijing, China; 3 Institute of Clinical Research, Odense University Hospital, Odense, Denmark; 4 Division of Pulmonary and Critical Care Medicine and Department of Pathology, University of Michigan, Ann Arbor, MI, USA; 5 Novartis Institutes for Biomedical Research, Cambridge, MA, USA; 6 Department of Biochemistry and Molecular Biology, University of Southern Denmark, Odense, Denmark

**Keywords:** Elastin, Extracellular matrix remodeling, Biochemical marker, Neoepitope, COPD, IPF, MMP

## Abstract

**Background:**

Elastin is an essential component of selected connective tissues that provides a unique physiological elasticity. Elastin may be considered a signature protein of lungs where matrix metalloprotease (MMP) -9-and -12, may be considered the signature proteases of the macrophages, which in part are responsible for tissue damage during disease progression. Thus, we hypothesized that a MMP-9/-12 generated fragment of elastin may be a relevant biochemical maker for lung diseases.

**Methods:**

Elastin fragments were identified by mass-spectrometry and one sequence, generated by MMP-9 and -12 (ELN-441), was selected for monoclonal antibody generation and used in the development of an ELISA. Soluble and insoluble elastin from lung was cleaved in vitro and the time-dependent release of fragments was assessed in the ELN-441 assay. The release of ELN-441 in human serum from patients with chronic obstructive pulmonary disease (COPD) (n = 10) and idiopathic pulmonary fibrosis (IPF) (n = 29) were compared to healthy matched controls (n = 11).

**Results:**

The sequence ELN-441 was exclusively generated by MMP-9 and -12 and was time-dependently released from soluble lung elastin. ELN-441 levels were 287% higher in patients diagnosed with COPD (p < 0.001) and 124% higher in IPF patients (p < 0.0001) compared with controls. ELN-441 had better diagnostic value in COPD patients (AUC 97%, p = 0.001) than in IPF patients (AUC 90%, p = 0.0001). The odds ratios for differentiating controls from COPD or IPF were 24 [2.06–280] for COPD and 50 [2.64–934] for IPF.

**Conclusions:**

MMP-9 and -12 time-dependently released the ELN-441 epitope from elastin. This fragment was elevated in serum from patients with the lung diseases IPF and COPD, however these data needs to be validated in larger clinical settings.

## Background

Elastin plays a critical role in the development of the cardiovascular, skin and respiratory system, as demonstrated when deletions and mutations in the elastic fibers result in supravalvular aortic stenosis (SVAS), William-Beuren syndrome (WBS) or cutis laxa (CL) [1,2]. SVAS and WBS are associated with increased vascular cell proliferation, narrowing of the aorta, peripheral pulmonary arteries, coronary and other major arteries, whereas CL results in an impaired vascular system and a severe dermal phenotype due to dermal inflammation and destruction of the elastic fibres [2,3].

The architecture of elastic fibres is tissue-specific reflecting the specific function of different tissues [4]. In general, elastic fibres are a major class of extracellular matrix molecules that are abundant in connective tissues. Elastic fibres are composed of elastin surrounded by a mantle of fibrillin-rich microfibrils. Elastin is formed by linking many soluble tropoelastin molecules catalyzed by lysyl oxidase, to create a massive insoluble, durable cross-linked array. Tropoelastin is characterized by hydrophobic mobile regions bounded by cross-links between lysine residues, referred as desmosine and isodesmosine, which stabilize the polymerized insoluble elastin and are essential for the elasticity [4].

In the lung, elastin fibres create a thin highly branched network throughout the respiratory tree to support the expansion and recoil of the alveoli during breathing. In the aorta and arteries, the elastin fibres are present in the medial layer, and form concentric fenestrated lamellae giving elasticity and resilience to the vessel walls [4]. Elastin fibres are very long-lasting with little turnover in healthy tissues [5]. However, various proteases such as matrix metalloproteinases (MMPs) and serine proteases are able to cleave elastin fibres by damaging the microfibrils and the elastin core [5-7], resulting in loss of elasticity. This loss of elasticity is a pathological feature of a number of degenerative and inflammatory diseases including vascular aneurysms [5,8] and chronic obstructive pulmonary disease (COPD) with co-existing emphysema [9,10]. For instance, deletion of the elastin gene in mice revealed lungs with emphysema-like lesions [11].

COPD is characterized by co-existence of emphysema, inflammation and narrowing in the small conducting airways and chronic changes in lung parenchyma which develop over many years. Idiopathic pulmonary fibrosis (IPF) is a progressive interstitial lung disease characterized by fibroblast proliferation and extracellular remodeling [12,13]. Lack of sensitive parameters of lung injury and destruction make quick evaluation of lung diseases difficult, which highlights the need for accurate and precise biochemical markers for diagnosis and prognosis, as well as early establishment of efficacy. Tools which have been suggested to indicate impaired physiological lung function, are computed tomography analysis and biochemical measurements of extracellular matrix degradation [14]. The pathogenesis of lung diseases such as COPD and IPF involves an inflammatory response [12,13], and tissue turnover is mediated in part by activated macrophages, which secrete their signature panel of proteases, including MMP-9 and -12 [12,13,15,16]. Desmosine and isodesmosine have been extensively discussed as potential indicators of elevated lung elastin fiber turnover, but their clinical validity and utility in urine and blood remains unproven. The major reasons are issues related to analytical validity of assays and lack of large longitudinal studies predicting progression and reflecting changes induced by effective treatment. These lysine residues are therefore still far from being considered as reliable biomarkers for COPD and IPF. [14,17,18]

Recently proteolytic generation of pathological- and tissue-specific fragments of proteins has received increased attention [19] as a potential marker of COPD and IPF. These protein fragments, referred to as neoepitopes or protein fingerprints [20,21], have proven to be more accurate predictors of disease than their unmodified intact protein origin [19]. For example, a type III collagen fragment generated by MMPs has been shown to be a marker for generalized and liver fibrosis [22,23], type II collagen degradation by MMPs has been demonstrated to be a marker for osteoarthritis and rheumatoid arthritis [24] and finally type I collagen fragments generated by cathepsin K, has been approved by the US Food and Drug Administration as a diagnostic tool for measuring and monitoring bone resorption [19].

Endopeptidases, such as MMPs, aggrecanases (ADAMTSs) and cathepsins, play a pivotal role in the degradation of extracellular matrix proteins in many diseases [25]. Especially MMP-9 and MMP-12 have been associated with elastin degradation and hence with cardiovascular [26] and respiratory diseases [15,16]. Our hypothesis was that elastin degradation by MMP-9 and -12, may provide information to aid diagnosis and progression of respiratory diseases.

The aims were to investigate the cleavage-type and kinetics of elastin and to develop an ELISA for quantitative assessment of MMP-degraded elastin. Finally the hypothesis was tested in a preliminary clinical setting investigating the discriminative diagnostic power.

## Methods

### In vitro cleavage of purified elastin from human tissue

Purified elastin from human aorta (Sigma Aldrich, prepared using the method described by Starcher et al. [27]) was cleaved with MMP-1, MMP-9, cathepsin K, cathepsin S (Calbiochem, VWR), MMP-3, MMP-8, MMP-12 (Abcam), ADAMTS-1, -4 and -8 (Abnova). The proteases were activated according to the manufacturers’s instructions. Each cleavage was performed separately by mixing 200 μg elastin/tissue and 2 μg of activated enzymes in MMP buffer (100 mM Tris–HCl, 100 mM NaCl, 10 mM CaCl_2_, 2 mM ZnOAc, pH 8.0), cathepsin buffer (50 mM NaOAc, 20 mM L-cystine, pH = 5.5) or aggrecanase buffer (50 mM Tris–HCl, 10 mM NaCl, 10 mM CaCl_2_, pH = 7.5). As the control, 200 μg elastin was mixed with MMP buffer only. The final concentration of elastin before cleavage was 0.33 mg/mL. Each aliquot was incubated for 2, 4, 24, 48, 72 and 169 hours at 37°C. All MMP cleavages were terminated using GM6001 (Sigma-Aldrich) and all cathepsin and aggrecanase cleavages using E64 (Sigma-Aldrich). Finally the cleavage was verified by visualization using the SilverXpress^®^ Silver Staining Kit (Invitrogen) according to the manufacturers’ instructions.

Using the same procedure as described above, purified elastin from non-soluble lung aorta (Sigma Aldrich, prepared using the method described by Starcher et al. [27]), soluble aorta and soluble lung (Sigma Aldrich, prepared using the method described by Partridge et al. [28]) were cleaved with MMP-9 and -12.

Human vascular tissue (atheroma-aorta, Biocat, Heidelberg, Germany) was cleaved by MMP-9 as described by Zhen et al. [25]. Digestion was carried out at 37°C by adding 1 μg activated MMP-9 in 250 μL digestion buffer (1 M Tris buffer (pH 7.4), NaCl, CaCl_2_, ZnOAc). Supernatants were removed on days 1, 3, 7 and 10 and frozen at −80°C. At each time point, MMP-9 in digestion buffer was added to the vascular wall sample after removing the supernatants and incubation was continued.

### Peptide identification by mass spectrometry

Analysis of the cleavage products of elastin purified from human aorta and of human vascular wall were performed in three different laboratories: A) Nordic Bioscience Beijing,China B) Department of Biochemistry and Molecular Biology at the University of Southern Denmark, Denmark, and C) as described by Zhen et al. [25]. The peptides were purified and desalted using reversed phase (RP) micro-columns (Applied Biosystems) prior to nanoLC-MS-MS analysis as described in the literature [29]. The purified peptides were re-suspended in 100% formic acid, diluted with H_2_O and loaded directly onto a 18 cm RP capillary column using a nano-Easy-LC system (Proxeon, Thermo Scientific). The peptides were eluted using a gradient from 100% phase A (0.1% formic acid) to 35% phase B (0.1% formic acid, 95% acetonitrile) over 43 min directly into an LTQ-Orbitrap XL mass spectrometer (Thermo Scientific). For each MS scan (Orbitrap), acquired t a resolution of 60000, 300–1800 Da range, the five most abundant precursor ions were selected for fragmentation (CID). The raw data files were converted to mgf files and searched in Mascot 2.2 software using Proteome Discoverer (Thermo Scientific). Peptides with a mascot probability score p < 0.05 were further analysed.

### Selection of peptide for immunizations

The first six amino acids of each free end of the sequences identified by MS were regarded as a neoepitope generated by the protease in question. All protease-generated sequences were analyzed for homology and distance to other cleavage sites and then blasted for homology using the NPS@: network protein sequence analysis [30].

### Immunization procedure

Six 4–6 week old Balb/C mice were immunized subcutaneously in the abdomen with 200 μL emulsified antigen (50 μg per immunization) using Freund’s incomplete adjuvant (KLH-CGG-VPGVGISPEA (Chinese Peptide Company, Beijing, China)). Immunizations were continued until stable titer levels were obtained. The mouse with the highest titer was selected for fusion and boosted intravenously with 50 μg immunogen in 100 μL 0.9% sodium chloride solution three days before isolation of the spleen for cell fusion. The fusion procedure has been described elsewhere [31]. The mouse work was approved by the Beijing laboratory animal administration office under approval number 200911250.

### Characterization of clones

The sequence VPGVGISPEA, named ELN-441, was selected for antibody generation. Native reactivity and peptide binding of the generated monoclonal antibodies were evaluated by displacement of human serum in a preliminary indirect ELISA using biotinylated peptides (Biotin-VPGVGISPEA) on a streptavidin-coated microtitre plate and the supernatant from the growing monoclonal hybridoma. Tested were the specificities of clones to the free peptide (VPGVGISPEA), a non-sense peptide, and the elongated peptide (VPGVGISPEAQ). Isotyping of the monoclonal antibodies was performed using the Clonotyping System-HRP kit (Southern Biotech). The selected clones were purified using Protein G columns according to manufacturer’s instructions (GE Healthcare Life Science).

### Assay protocol

The selected monoclonal antibody was labeled with horseradish peroxidase (HRP) using the Lightning link HRP labeling kit according to the instructions of the manufacturer (Innovabioscience). A 96-well streptavidin plate was coated with 0.4 ng/mL Biotin-VPGVGISPEA dissolved in assay buffer (25 mM Tris, 1% BSA, 0.1% Tween-20, pH 7.4) and incubated for 30 minutes at 20°C. 20 μL of free peptide calibrator or sample were added in duplicate to appropriate wells, followed by 100 μL of conjugated monoclonal antibody and incubated for 1 hour at 20°C. Finally, 100 μL tetramethylbenzinidine (TMB) (Kem-En-Tec) was added and the plate was incubated for 15 minutes at 20°C in the dark. All the above incubation steps included shaking at 300 rpm. After each incubation step the plate was washed five times in washing buffer (20 mM Tris, 50 mM NaCl, pH 7.2). The TMB reaction was stopped by adding 100 μL of stopping solution (1% HCl) and measured at 450 nm with 650 nm as the reference. A master calibrator prepared from the synthetic-free peptide accurately quantified by amino acid analysis was used as a calibration curve and plotted using a 4-parametric mathematical fit model.

### Technical evaluation and specificity

From 2-fold dilutions of quality control (QC) serum and plasma samples, linearity was calculated as a percentage of recovery of the 100% sample. The lower limit of detection was determined from 21 zero serum samples (i.e. buffer) and calculated as the mean+3X standard deviation. The inter- and intra-assay variation was determined by 12 independent runs of 8 QC serum samples, with each run consisting of two replicas of double determinations. The stability of serum was measured using three serum samples, which were frozen and thawed between one and 10 times.

The antibody ELN-441 was evaluated using the materials described under “In vitro cleavage”, where elastin was cleaved by different MMPs, cathepsins and aggrecanases. The samples were diluted 1:10 in the ELISA.

### Clinical validation of ELN-441

ELN-441 levels were measured in serum from patients diagnosed with COPD (n = 10) and IPF (n = 29) and compared with controls (n = 11). The COPD and IPF serum samples were obtained as a part of the “lung tissue research consortium” ( http://www.ltrcpublic.com). The local IRB evaluated the study and concluded that due to the proper de-identification of samples and patients by the LTRC, an approval from the IRB was not required for this work. The controls were derived from a previously described study [32,33]. The samples were diluted 1:2 in the ELN-441 assay.

### Statistics

Serum levels of ELN-441 in COPD/IPF patients and controls were compared using two-sided non-parametric Wilcoxon rank sum test. Area under the curve was calculated using the Receiver Operating Characteristic (ROC). The likelihood of patients having ELN-441 was investigated as an odds ratio, extrapolated from weighted levels, with the lowest value in the population being set at 0 and the highest at 1, and all subjects classified as having normal (within the 1.8xSD + mean of the normal population) or high (>1.8xSD + mean) levels of the biomarker. Results were considered statistically significant if p < 0.05.

## Results

### Analysis of protease generated elastin degradation

Analysis of cleavage sites of purified elastin from human aorta is shown in Table 1. A total of 114 identified different fragments were generated: 6 by MMP-1, 7 by MMP-3, 11 by MMP-8, 4 by MMP-9, 10 by cathepsin K, 12 by cathepsin S, 24 by ADAMTS-1, 19 by ADAMTS-4 and 21 by ADAMTS-8. The majority (73%) of the cleavages involved alanine (A), valine (V) or glycine (G). Glycine was involved in most (40%) of the cleavages. Half of the amino acids involved in the cleavage sites were hydrophobic (47%) and the other half hydrophilic (53%), however most cleavages of hydrophobic amino acids took place at the amino acids NH_2_-group (67%) and opposite for the hydrophilic amino acids (73%).

**Table 1:** Analysis of the individual amino acids involved in the cleavages of elastin

**Hydrophobocity**	**Amino acid type**	Share of elastin*	**No. of cleavages in the N-terminal**		**No. of cleavages in the C-terminal**		**Percentage of cleavages**		
			**R-COOH↓**	**↓NH**_**2**_**-R**	**R-COOH↓**	**↓NH**_**2**_**-R**	**R-COOH↓**	**↓NH**_**2**_**-R**	**Total**
Hydrofobic	**A**	21%	15	30	16	36	14%	29%	21%
	**P**	13%	5	5	4	4	4%	4%	4%
	**V**	12%	5	37	2	12	3%	22%	12%
	**L**	7%	8	5	3	2	5%	3%	4%
	**I**	2%	–	3	–	1	<1%	2%	1%
	**F**	2%	4	3	–	15	2%	8%	5%
	*Total*	*57%*					*27%*	*67%*	*47%*
Hydrophilic	**G**	28%	71	22	56	33	56%	24%	40%
	**K**	4%	3	–	21	–	11%	<1%	5%
	**S**	2%	–	–	–	2	<1%	1%	<1%
	**Y**	2%	1	3	–	2	<1%	2%	1%
	**T**	2%	1	1	–	5	<1%	3%	2%
	**R**	2%	–	4	12	–	<1%	2%	4%
	**Q**	1%	–	–	–	2	<1%	1%	<1%
	*Total*	*43%*					*73%*	*33%*	*53%*

114 different peptides were identified. Two cleavage sites (one in the N-terminal and one in the C-terminal) are involved in generating each of these peptides. Each of these cleavage sites involve two amino acids (one cleaved at the NH_2_-group and the other one at the COOH-group). Arrow (**↓**) indicates the cleavage site.

*The content of M, E, D, H and C in elastin are ≤1% and not included in the table.

**Figure 1:**
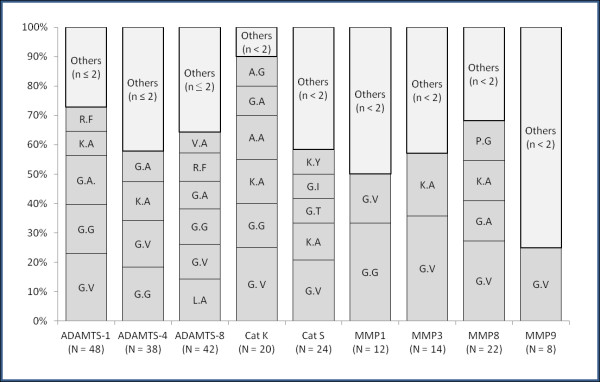
**The distribution of type of cleavage sites in the presence of various proteases.** N indicates number of total cleavage sites for each protease and n indicates number of type of cleavage site.

Cleavages between glycine-valine and glycine-alanine were predominant in the N-terminal of the identified peptides, whereas glycine-glycine and lysine-alanine were favored in the C-terminal end of the peptides (data not shown). Glycine-valine cleavages were created by all the proteases but more commonly by MMPs. The glycine-glycine cleavage site most frequently involved MMP-1 (Figure 1). ADAMTS-8 was the only protease to cleave between leucine-alanine, while arginine-phenylalanine cleavages were only produced by ADAMTS-1 and -8 (Figure 1). Lysine-alanine and glycine-alanine cleavages were shared among the proteases.

### Selection of the most promising neoepitope

A selection of the cleavage kinetics of MMP-9 and -12 generated fragments analysed by laboratory B, is illustrated in Table 2. A total of 416 different peptides were identified of which 132 were identified in elastin preparations with no added proteases. Some of the peptides were only generated by one of the MMPs, others by both MMP-9 and -12. The time of digestion varied with some peptides being generated immediately, others after days of incubation, and some peptides continued to be degraded with subsequent incubations.

**Table 2:** Examples of cleavage kinetics of MMP-9 and -12 generated peptides identified by MS in elastin from human aorta

**Amino acid no.**	**Identified peptides in elastin from aorta**	**MMP-9 cleaved (hours)**						**MMP-12 cleaved (hours)**				
		**0**	**2**	**24**	**48**	**72**	**≥169**	**2**	**24**	**48**	**72**	**≥169**
041-051	VFYPGAGLGAL					x	x					
041-057	VFYPGAGLGALGGGALG		x	x	X	x	x					
041-059	VFYPGAGLGALGGGALGPG		x	x	X							
085-102	VTFPGALVPGGVADAAAA									x	x	
141-159	VPGVGLPGVYPGGVLPGAR	x	x	x	X	x	x	x	x	x	x	x
158-164	ARFPGVG		x									
229-241	GYGPGGVAGAAGK									x	x	x
230-241	YGPGGVAGAAGK		x	x	X	x		x	x		x	
280-293	AGVPGVPGAIPGIG			x	X			x	x	x	x	x
281-294	GVPGVPGAIPGIGG						x	x	x	x	x	x
302-312	AAAAAAAAAAK							x				
327-339	PGFGPGVVGVPGA		x	x				x	x			
384-408	GARPGVGVGGIPTYGVGAGGFPGFG		x	x	X	x			x			
385-392	ARPGVGVG	x	x	x								

The length of the identified protease-generated peptides was between 10 and 45 amino acids. They were tested for homology and cross-reactivity to other proteins to select sequences that were unique and the most representative of elastin degradation. The sequence selected was VPGVGISPEA↓ since it was identified by LC-MS/MS in in vitro MMP-9 and -12 cleaved elastin purified from human aorta (Table 3) and was also identified in MMP-9 digested elastin from the vascular wall (laboratory C) (Table 4). The sequence VPGVGISPEA↓ was also identified in a single peptide generated by MMP-1. The sequence had a very conservative C-terminal and was found in more than one peptide. The sequence was named ‘ELN-441’ due to the cleavage site at alanine with amino acid number 441. ELN-441 was selected for immunization and antibody generation.

**Table 3:** Peptides cleaved at amino acid no. 441 identified by MS in elastin purified from human aorta cleaved in vitro by MMP-9 and -12

**Amino acid no.**	**Identified peptides in elastin from aorta**	**MMP-9 cleaved (hours)**					**MMP-12 cleaved (hours)**				
		**2**	**24**	**48**	**72**	**≥169**	**2**	**24**	**48**	**72**	**≥169**
423-441	VGGVPGVGG**VPGVGISPEA**					x					
425-441	GVPGVGG**VPGVGISPEA**		x			x				x	x

**Table 4:** Peptides cleaved at amino acid no. 441 identified by MS in MS in human vascular wall cleaved in vitro by MMP9

**Amino acid no.**	**Identified peptide in vascular wall**	**MMP-9 cleaved (hours)**			
		**24**	**72**	**168**	**240**
409-441	VGVGGIPGVAGVPSVGGVPGVGG**VPGVGISPEA**			x	
417-441	VAGVPSVGGVPGVGG**VPGVGISPEA**			x	x
422-441	SVGGVPGVGG**VPGVGISPEA**				x
423-441	VGGVPGVGG**VPGVGISPEA**			x	

### Assay development and validation

**Figure 2:**
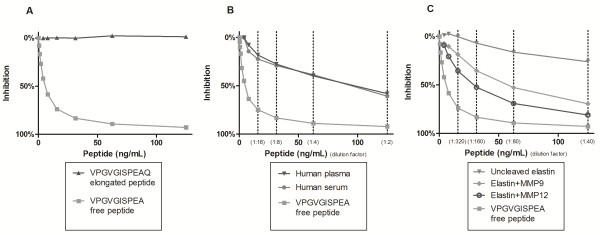
**Characterization of the ELN-441 monoclonal antibody.** ELISA showing percent inhibition of the signal of: A) the free peptide and elongated peptide, B) the free peptide and the native human serum and plasma samples which were run diluted 1:2, 1:4 and so forth as indicated by the dotted lines, C) the in vitro cleaved elastin from aorta with and without MMP-9 and -12. The materials were run diluted 1:40, 1:80 and so on as indicated by the dotted lines.

The requirements for selecting the monoclonal antibodies for ELISA development were I) IgG subtype, II) specificity towards the neoepitope and not the elongated peptide or uncleaved elastin, III) native reactivity towards diseased human body fluids and not only to the synthetic peptide and IV) acceptable dilution recoveries in human body fluids. Based on these requirements an antibody recognizing the sequence VPGVGISPEA was selected. The monoclonal antibody did not show any affinity toward either the elongated peptides or the uncleaved elastin (Figure 2A and C). The native reactivity towards diseased human serum and plasma was high and the signal was almost inhibited completely (Figure 2B). These findings were consistent in repeated batches.

The results of the technical evaluation of the assay known as “ELM” are in Table 5 and show a technically robust assay with dilution recovery within the recommended range of +/−10%. The accuracy and precision was acceptable with low inter- and intra-assay variation.

**Table 5:** Summary table of the technical validation of ELM

**Technical validation step**	**ELN-441**
Target	MMP degradation of human elastin
Detection range	0,484–125 ng/mL
Dilution range of serum samples	1:2, 1:3 and 1:4 is recommended
Dilution range of plasma samples	1:2, 1:3 and 1:4 is recommended
Dilution recovery of human serum*	91%
Dilution recovery of human plasma*	95%
Intra-assay variation**	9.44%
Inter-assay variation**	13.8%
Analyte stability***	103%

*Percentage dilution recovery was calculated as the mean of 5 human samples diluted 1:2 and 1:4. **Inter- and intra-assay validation was calculated as the mean variation between 8 individual determinations of each human serum sample. ***The stability of the analyte (human serum) was calculated as the mean of three different serum samples were tested after freeze/thaw for one to 10 times.

### Generation of ELN-441 is dependent on cleavage time and solubility of tissue

**Figure 3:**
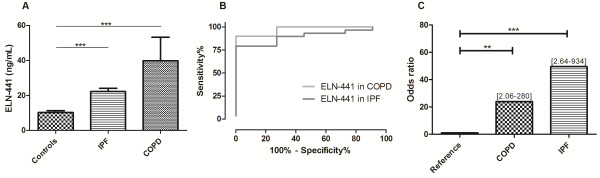
**Biological validation of ELN-441 in human serum from patients with COPD (n = 10) and IPF (n = 29) compared with controls (n = 11).** A) Bars indicate mean level. Groups were compared by Wilcoxon rank sum test. B) ROC curve; AUC_COPD_ = 97% and AUC_IPF_ = 90%. C) Odds ratio. Data are shown as mean ± 1.8SD with 95% confidence intervals.

By LC-MS/MS analysis the sequence VPGVGISPEA was identified as being generated by MMP-9 and -12, and from the ELISA characterization it was confirmed that these MMPs were able to generate the fragment in amounts high enough to be detected by the ELISA (Figures 2C and 3). The cleavages were observed to be dependent on the solubility of the tissue and the type of protease. MMP-9 and -12 generated equal amounts of ELN-441 when added to soluble lung (Figure 3A), but when added to insoluble lung only MMP-12 was able to generate ELN-441 and in much lower quantities (Figure 3B). The cleavage fragment was released in a time-dependent manner as seen in (Figure 3C).

**Figure 4:**
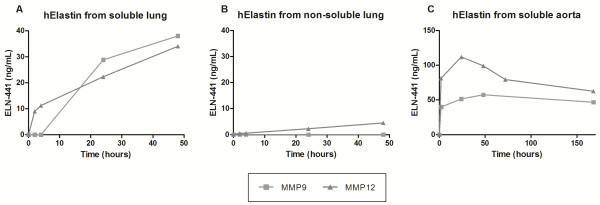
**Release of ELN-441 by MMP-9 and -12 cleavages as a function of time of human elastin from different tissues: A) soluble elastin, B) non-soluble elastin and C) soluble aorta.** The cleaved material was diluted 1:10 in the assay.

### ELN-441 is elevated in patients with COPD and IPF

Levels of the MMP-9 and MMP-12 generated neoepitope ELN-441 were significantly higher in serum from patients diagnosed with COPD (p < 0.0003) and with IPF (p < 0.0001) compared with controls (Figure 4A).

### Diagnostic value of ELN-441 to differentiate between COPD and IPF patients and controls

To investigate the diagnostic value of ELN-441, the ROC curves were produced and the area under the curve (AUC) calculated (Figure 4B). ELN-441 had the best diagnostic value in COPD patients (AUC 97%, p = 0.00025), with lower diagnostic value in IPF patients (AUC 90%, p = 0.00011). The odds ratios (Figure 4C) for differentiating controls from COPD and from IPF patients indicate that COPD diagnosing had the highest value (24, [2.06–280]) compared with IPF diagnosing (50, [3.64–934]). The controls were normally distributed and the upper limit of normal was mean+1.8xSD.

## Discussion

This study provides the following important information on the diagnosis and progression of important lung diseases:

1) Elastin is degraded by different proteases at different times. This degradation pattern adds to information already described by others [34-36]. We selected a specific neoepitope as a candidate novel biomarker of lung disease, and developed an ELISA (ELM) for quantifying this unique target. To our knowledge, this assay is the first to quantify MMP degradation of elastin, in both in vitro generated material and in human fluids. This tool may provide value for other researchers and for the characterization of patients.

2) The ELN-441 neoepitope was generated by MMP-9 and -12 in a time dependent manner for soluble elastin, while in the case of insoluble elastin, only MMP-12 was able to generate the fragment.

3) The selected neoepitope of elastin was based on our MS analysis cleavage pattern by MMP-12, which is the protease known to be highly expressed in macrophages during lung inflammation.

4) By preliminary analysis in a limited number of patients, the ELM degradation marker exhibited highly specific diagnostic power, in particular for COPD with an AUC over 97%, and IPF with an AUC over 90%.

This study showed that elastin was degradable by MMPs, cathepsins as well as aggrecanases. The bulk of the identified peptides from in vitro cleaved soluble elastin was however generated by aggrecanases. This is an important observation as a recent publication highlighted the aggrecanases as important molecules in lung diseases [37], and the fact that different proteases have different molecular characteristics [38]. It is well appreciated in cartilage pathologies that aggrecanase and MMP mediated cartilage destruction provide different molecular information [49]. Due to the sensitivity of the MS–technology, we identified elastin in the non-proteolytical fraction that was degraded in vitro. Whether this may indicate hot-spots for protein degradation, instability or artifacts during the purification procedure remains to be investigated. Interestingly, different numbers and fragments of identified peptides were obtained in the three different MS-laboratories. This may reflect different equipment and emphasizes the fragility of the current approach, and the necessity for cross-validation by multiple runs of the identified fragments in different cleavages by different equipments.

Although elastin fragments generated by aggrecanases and cathepsins were identified and may serve as biomarker targets for other indications, our study focused on the activity of MMP-9 and -12 because these proteases are expressed in respiratory diseases [15,16]. To some extent the two MMPs have a similar degradation profile, cleaving at many of the same sites, although some unique sites were also identified. Measuring the release of ELN-441 from elastin-rich tissues emphasized that only MMP-12, and not MMP-9, is able to degrade elastin from insoluble lung and that MMP-12 is faster to degrade elastin from aorta than MMP-9.

Of the elastin cleavages, 73% involved alanine, valine and glycine, of which glycine was predominant. This was as expected, alanine, valine and glycine compose 3 out of the four main amino acids in elastin. The fourth amino acid is proline. Alanine, valine and glycine are the amino acids with the shortest molecular chain leading to the smallest steric hindrance and probably easy accessible. This might be the reason for the reduced cleavage at proline, since it contains a pyrrolidin ring. Half of the amino acids making up elastin are hydrophobic which matches the outcome that half of the amino acids involved in the cleavage of elastin are hydrophobic. Interestingly the majority of the cleavages of the hydrophobic amino acids took place at the NH_2_-group of the amino acid. The opposite was observed for the hydrophilic amino acids in which the COOH-group was the preferred cleavage site. Cleavage sites involving glycine specially glycine-valine and glycine-glycine are not protease specific since aggrecanases, cathepsins and MMPs recognize these sites. Aggrecanases differ from the other proteases by degrading elastin at different cleavage sites such as between leucine-alanine and arginine-phenylalanine. Aggrecanase generated neoepitopes may therefore have a different diagnostic profile than for example MMPs. The cleavage products are dependent on incubation time, amount of protease and the stability of the peptide, as observed by others [40].

Elastin degradation has been investigated by several groups [14,41-45] conducting analyses of the cleavage pattern and of proteases involved as a consequence of inflammation and macrophage activity. When analyzing the MMP-12 degradation of tropoelastin Taddese et al. and Heinz et al. both identified the ELN-441 fragment [34,36]. Barroso et al. did not identify ELN-441, but observed that the amount of degradation peptides is highly related to the amount of protease [40]. Furthermore, it has been shown that elastin degradation fragments, in particular a MMP-12 generated repeated sequence fragment, acts as a chemo-attractant for monocytes and fibroblasts in vitro [41,42] and that autoimmune response to elastin fragments has been identified [46].

A battery of proteases, in particular MMPs, has been shown to be important mediators in lung disease. MMP and neutrophil elastase expression was investigated in patients with COPD and healthy controls using bronchoalveolar lavage fluid to analyse macrophage expression of the different MMPs [47]. It was found that MMP-9, MMP-8, and MMP-1 along with neutrophil elastase were significantly increased in COPD patients compared with healthy controls. Another group also showed that MMP-12 is necessary for macrophage recruitment in the lungs of smoke-exposed mice since MMP-12 knockout mice failed to develop inflammation in response to cigarette smoke [48]. Interestingly, a study investigated the ability of human and mouse monocyte-derived macrophages to degrade elastin ex vivo, concluded that MMP-12 may not be an elastolytic enzyme but is rather an inducer of an unknown pathway that activates elastin-degrading enzymes [49]. These data are in contrast to our in vitro data clearly showing that insoluble elastin may be degraded by MMP-12 but not MMP-9. The role of MMP-9 in cigarette smoke-induced COPD was investigated in study including MMP-9 over-expressing mice, MMP-9 knockout mice, and in patients that had undergone lung transplantation [50]. Data showed that MMP-9 expression was not correlated to severity of disease, albeit in the mouse models an integrated part of the disease. Our findings show that MMP-9 was not able to generate the ELN-441 fragment from insoluble elastin, when assessed using the ELM assay.

In the present study we identified the ELN-441 biomarker as diagnostically sensitive for COPD and IPF, as compared to controls. This suggests that the hypothesis stating that lung destruction is driven by MMP-9 and MMP-12 is valid and can be quantified. The diagnostic power of ELN-441 was higher for COPD than IPF, which is in accordance with the recent research in the field of MMPs and elastin in COPD, and that the main pathologic problem in IPF might be pulmonary fibrosis and not elastin degradation. However, one complicating factor in the use of ELN-441 is that elastin expression is not restricted to the lung tissues, as arteries, skin and tendons have been shown to express this protein [4]. Thus, several co-morbidities may influence the systemic levels of ELN-441. Further investigations are needed to determine each tissue’s contribution to the total pool of the ELN-441 neoepitope, and possibly other ELN epitopes.

One major limitation of the current clinical study of ELN-441 was the relatively small sample size and the limited clinical information obtained. Thus these preliminary findings need to be validated in larger clinical settings for the diagnostic utility, and also for prognostic potential.

Other researchers have investigated an array of biomarkers in induced sputum, exhaled air condensate, bronchial biopsy, bronchoalveolar lavage fluid, urine and peripheral blood that could be used as diagnostic and prognostic tools for lung diseases [51]. There is, however, a relative lack of information about how these biomarkers relate to disease severity and to other disease measurements such as FEV1, how reproducible they are, and how they may be affected by therapies. Desmosine and isodesmosine have been extensively discussed as biomarkers of elastin turnover since they are unique to human elastin. ELISA measurements of desmosine and isodesmosine in serum have, however, been shown to be incapable of discriminating between normal and COPD subjects [14]. Others have investigated serological biomarkers of the elastin-derived peptides (EDPs), which have been found elevated in plasma of patients with COPD, but it is unclear whether these peptides reflect elastin turnover in the lung or in other compartments of the body such as the arteries [5,10]. Nevertheless, a correlation between EDPs and lung damage on computed tomographic scans has been shown [9]. EDPs are detected with use of polyclonal antibodies making the method less sensitive than assays using monoclonal antibodies. Several other serological biomarkers have been investigated such as; fibrinogen, C-reactive protein, Trolox equivalent antioxidant capacity, CXCR2, TGF-beta, TNF-α [51] and Clara cell secretory protein-16 [52]. Of these, only C-reactive protein and TNF-α showed a relationship with FEV1-based disease staging criteria of COPD in a meta-analysis by Franciosi et al. [53]. However the separation was small, demonstrating poor sensitivity and diagnostic potential. Ultimately, a panel of biomarkers may be needed to characterize different aspects of lung disease in patients, and for prognosis, diagnosis and assessment of efficacy of intervention.

The neoepitope technology, measuring of specific protein degradation fragments, allows for assessment of specific proteolytic activity in given tissues, provided that the sequence is unique for one or fewer proteases. The present assay quantifies the peptide in elastin which is cleaved at the 441 position, and not the elongated peptide containing an extra amino acid at the cleavage site, nor intact elastin. Thus, this assay allows for quantification of one specific sub-pool of the elastin molecule—namely the soluble, degraded one. The sequence was MMP specific, whereas other fragments identified seems to be specific for aggrecanases and cathepsins. Other assays will have to be developed to allow the quantification of these epitopes, providing different biological or pathological information.

In conclusion, a robust assay has been developed using a specific monoclonal antibody for detection of ELN-441, a MMP-9 and -12 generated fragment of elastin. It was demonstrated that this fragment was significantly elevated in COPD and IPF patients and has high diagnostic potential. Further, larger, clinical studies are needed to confirm the diagnostic value and also to evaluate the prognostic potential in lung disease, and the potential utility of this neoepitope in other diseases in which elastin degradation may be a pivotal pathological feature.

## Abbreviations

ADAMTS: A disintegrin and metalloproteinase with thrombospondin motifs; AUC: Area under the curve; CL: Cutis laxa; COPD: Chronic obstructive pulmonary disease; CXCR2: Interleukin 8 receptor beta; EDP: Elastin derived peptides; ELISA: Enzyme-linked immunosorbent assay; ELM: Elastin cleaved by matrix metalloproteinase; ELN: Human elastin; FEV1: Forced expiratory volume in 1 second; HRP: Horse radish peroxidase; IPF: Idiopatic pulmonary fibrosis; LC-MS: Liquid chromatography mass spectrometry; MMP: Matrix metalloproteinase; MS: Mass spectrometry; QC: Quality control; ROC: Receiver operating characteristic; SD: Standard diviation; SVAS: Supravascular aortic stenosis; TGF-beta: Transforming growth factor beta; TNF-alpha: Tumour necrosis factor alpha; TMB: Tetramethylbenzinidine; WBS: William-Beuren syndrome.

## Competing interests

Morten Karsdal holds stock in Nordic Bioscience. Lloyd Klickstein holds equity in Novartis AG. Other authors have no competing interests.

## Authors’ contribution

HSA, MRL and AN did the peptide identification and selection. HSA, REC, QHTN, YW and QZ were involved in the assay development of the new marker, while FJM, CMH, MH and LBK provided the clinical samples. HSA, DJL and MAK contributed in the process of idea to product and also in writing the article.

## Pre-publication history

The pre-publication history for this paper can be accessed here:

http://www.biomedcentral.com/1471-2466/12/34/prepub
